# Genotype Analysis as a Clinical Tool for Families in Hypertrophic Cardiomyopathy∗

**DOI:** 10.1016/j.jacadv.2023.100605

**Published:** 2023-09-06

**Authors:** Mark V. Sherrid, Daniele Massera

**Affiliations:** Hypertrophic Cardiomyopathy Program, Division of Cardiology, NYU Langone Health, New York City, New York, USA

**Keywords:** family counseling, gene therapy, genetics, genotype, hypertrophic cardiomyopathy, preimplantation genetic testing, prognosis

**Figure:**
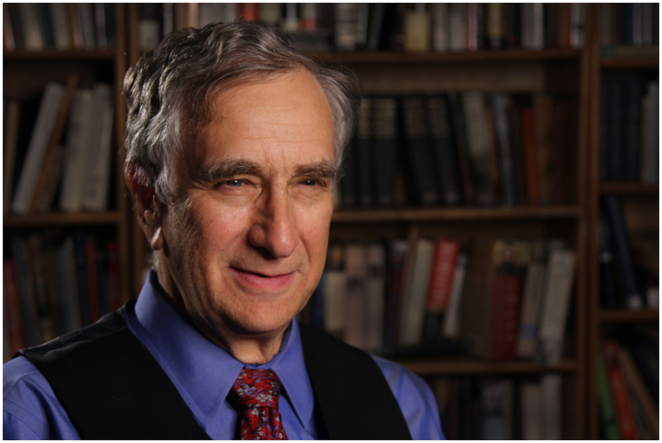

The effort by Nielsen et al^1^ reported in this issue of *JACC: Advances* is admirable on several levels. First, 90% of invited hypertrophic cardiomyopathy (HCM) index patients accepted the investigators’ invitation to have genotype analysis. Of the 903 relatives of the 183 index patients, 84% accepted an invitation to participate in the now-reported study of imaging penetrance to the HCM phenotype and its clinical consequences. This leaves these editorialists applauding from afar the cooperation and trust of these patients and their families. Moreover, this commitment of families and investigators persisted over 8 years of follow-up to provide a comprehensive dataset detailing the incidence of clinically important events.

In patients with a pathogenic/likely pathogenic variant (P/LP genotype-positive), penetrance on echocardiography or cardiac magnetic resonance was 39% at age 43 ± 18 years. Penetrance was higher in men than in women and greater in those over 50 years of age. Symptoms at HCM diagnosis were more common in patients than in relatives (70% vs 45%). The incidence of major adverse cardiac events defined here as a composite of disabling stroke, transplant, death from heart failure, and sudden cardiac death (SCD) was 1.2%/y in the genotype-positive relatives with phenotypic HCM, not different from 1.5%/y in the index patients, over 8 years of follow-up. Anatomic location of HCM did not follow suit from index patient to relative.

This data answers several questions that patients at our HCM Program ask about our recommendation to clinically screen first-degree relatives with echocardiography and electrocardiogram and also to genotype the index patient. The patients ask how this will help our relatives. What will it mean if they are found to have HCM or my abnormal variant?

In the Sarcomeric Human Cardiomyopathy Registry, patients with P/LP sarcomere variants had a 2-fold greater risk for adverse outcomes, mainly heart failure, compared with patients without mutations; sarcomere variants of uncertain significance were associated with intermediate risk.^2^ The data from Nielson et al likewise highlights that there is also a risk for gene-positive relatives who develop HCM. While 1.2%/y major adverse cardiac event may not seem alarming for a 70-year-old, the cumulative effect over the decades for affected relatives with an average age of 43 years focuses attention. However, though family cascade screening in genotype-positive families has utility for identifying relatives at risk for developing clinical HCM and its sequelae, the therapeutic benefit for the index patient has remained elusive at least until 2023.^3^, ^4^, ^5^

While the prevalence of symptoms was lower in the gene-positive relatives than in the index patients, experience has shown incremental symptoms over ensuing years. Treatment falls into at least 3 domains given the complexity of HCM expression: sudden death prevention, treatment of limiting symptoms, and stroke prevention from atrial fibrillation.

Atrial fibrillation occurred in 15% of the gene-positive relatives, highlighting the importance of surveillance for arrhythmia in the relatives with left ventricle thickening, with periodic prolonged monitoring and anticoagulation when clinically appropriate. Sudden death risk has not differed between the genotype-positive and genotype-negative groups. As such, identification of an HCM-associated variant is not included in recommendations for implantable cardioverter-defibrillator (ICD) implantation in America or Europe.^4^^,^^5^

Twenty-one relatives died suddenly or had an appropriate ICD discharge. There is controversy over the best clinical scheme for identifying patients at high risk for SCD. For US experts, presence of one major clinical risk factor prompts at least discussion and consideration of ICD implantation in younger (<60 years) patients.^4^, ^5^, ^6^ In contrast, European guidelines rely on the European Society of Cardiology risk score. Experts from Boston have contested its utility by demonstrating lack of sensitivity for detecting patients at high risk who ultimately have SCD or appropriate discharges.^6^ In the present study, this lack of sensitivity was demonstrated in the 18 gene-positive patients who died suddenly under observation (10 index patients and 8 relatives): 11 (61%) had European Society of Cardiology risk scores <4%. We don’t know how they would have been managed under the scheme favored by the current American College of Cardiology/American Heart Association guidelines.

Will there eventually be effective gene therapy for HCM, and will it be widely applied? Two recent studies using gene editing bring hope in this regard.^7^^,^^8^ In both, an adenine base editor and single guide RNA system encapsulated in AAV9 vectors were injected into newborn mice; the HCM genotype was corrected in 30% to 60% of cardiomyocytes with subsequent prevention of hypertrophy and fibrosis. Such studies have not yet been performed in mice that already have an established phenotype. A trial of gene therapy for patients with HCM due to haploinsufficiency of myosin-binding protein C3 is under consideration by the United States Food and Drug Administration (NCT05836259); this effort has been fast-tracked by the US FDA and has received orphan drug designation.

There is latency during childhood and often in young adult life before left ventricle hypertrophy occurs in a genotype-positive individual. This offers a potential time window to consider treatment. Questions arise about whether such efforts are appropriate or will be cost-effective for HCM patients or their offspring, considering that most patients with HCM live to normal life expectancy, and that those more severely affected have recourse to therapies that both prevent sudden death and palliate or resolve symptoms.^4^ Moreover, though the numbers of genes that have been associated with HCM are less than a score, the numbers of point mutations are >1,000. This would truly be personalized medicine. Moreover, it would not include the ∼50% to 60% of patients without a detected associated variant. Downstream functional and structural targets might (or might not) prove to be more widely applicable.^9^

As with many therapies in clinical medicine, the question may turn out to be not how to treat but who to treat. The above-mentioned experiments in mice were performed in the myosin heavy chain mutation p.Arg403Gln, whose French-Canadian family was the first genotype identified, studied because of its severe and early penetrance.^10^ Such a family might have little hesitation. In this regard, there has been a recent effort to define the risk of MYBC3 P/LP variants in children (NCT05112237).

In contrast to gene editing strategies that are largely in the basic science stage, preimplantation genetic testing for monogenic conditions (PGT-M) is currently an ongoing clinically applied tool.^11^^,^^12^ PGT-M is a genetic test performed on embryos created through in vitro fertilization (IVF) that is designed for prospective parents who know they are at increased risk of having a child with a specific genetic disorder to prevent passing the P/LP variant to the next and subsequent generations. Single cells from IVF-generated embryos are tested for the P/LP variant after polymerase chain reaction amplification. Embryos without the variant are implanted in the mother’s uterus; those with the variant are not. Its advantages are that it is currently available with minimal risk to the mother and a very high chance of a successful, disease-free newborn. Disadvantages include a maternal risk from hormonal stimulation for development of multiple eggs in the ovary and a procedural risk from egg retrieval (both relatively low), discomfort from egg retrieval, and a high cost (above that of standard IVF) not covered by insurance. At our institution, we have recommended this approach to several families with severely impactful, genotype-positive HCM who have ultimately had genotype-negative infants. As far as we know, there has not yet been a comprehensive registry of PGT-M results in HCM families. With such a registry, the success rate and maternal morbidity of this strategy could be ascertained and then communicated. Clearly, such a registry would need to include infant genotypes to assure successful embryo selection was achieved. The wish to protect one’s children from a disabling and potentially lethal condition is strong; efforts such as Nielson et al underscore the motivation.

## Funding support and author disclosures

Dr Massera has done consulting for Tenaya Therapeutics, Sanofi, and Chiesi. Dr Sherrid has reported that he has no relationships relevant to the contents of this paper to disclose.
